# [Retracted] MicroRNA-429 inhibits gastric cancer migration and invasion through the downregulation of specificity protein 1

**DOI:** 10.3892/ol.2026.15466

**Published:** 2026-01-19

**Authors:** Jingbin Ni, Yisha Yang, Di Liu, Hui Sun, Shimao Jin, Jingying Li

Oncol Lett 13: 3845–3849, 2017; DOI: 10.3892/ol.2017.5869

Following the publication of this paper, it was drawn to the Editor's attention by a concerned reader that, for the cell migration and invasion assay data shown in Fig. 2 on p. 3487, the ‘miR-429 / SGC-7901’ invasion panel appeared to show an overlap with the ‘miR-429 / AGS migration’ panel; moreover, the ‘NC / AGS migration’ panel appeared to show an overlap with the ‘NC / AGS invasion’ panel, suggesting that data which were intended to show the results of differently performed experiments had been derived from the same original sources. A subsequent analysis of the data in this paper performed by the Editorial Office revealed that these data were strikingly similar to data that had appeared in Fig. 4 of another paper written by different authors at different research institutes that was received a small number of weeks later at the same journal (*Oncology Letters*), which has since been retracted on account of its apparent sharing of data with other articles.

Owing to the number of overlapping data panels identified in Fig. 2, and given the fact that the contentious data in the above article were submitted for publication at around the same time in another paper that has since been retracted, the Editor of *Oncology Letters*, has decided that this paper should be retracted from the Journal. The authors were asked for an explanation to account for these concerns, but the Editorial Office did not receive a reply. The Editor apologizes to the readership for any inconvenience caused.

